# Predictors of nurses’ and midwives’ intentions to provide maternal and child healthcare services to adolescents in South Africa

**DOI:** 10.1186/s12913-016-1901-9

**Published:** 2016-11-15

**Authors:** Kim Jonas, Priscilla Reddy, Bart van den Borne, Ronel Sewpaul, Anam Nyembezi, Pamela Naidoo, Rik Crutzen

**Affiliations:** 1 School of Public Health and Primary Care (CAPHRI), Department of Health Promotion, Maastricht University, PO Box 616, 6200 MD Maastricht, A Peter Debyeplein 1, 6229 HA Maastricht, The Netherlands; 2 Human Sciences Research Council (HSRC), Population Health, Health Systems and Innovation Unit, Cape Town, South Africa; 3 Faculty of Community and Health Science, University of the Western Cape, Cape Town, South Africa

**Keywords:** Adolescents, Attitude, Family planning, Knowledge, Intention, Maternal healthcare, Midwife, Nurse, Subjective norms

## Abstract

**Background:**

Adolescent mothers are at a much higher risk for maternal mortality compared to mothers aged 20 years and above. Newborns born to adolescent mothers are also more likely to have low birth weight, with the risk of long-term effects such as early onset of adult diabetes than newborns of older mothers. Few studies have investigated the determinants of adequate quality maternal and child healthcare services to pregnant adolescents. This study was conducted to gain an understanding of nurses’ and midwives’ intentions to provide maternal and child healthcare and family planning services to adolescents in South Africa.

**Methods:**

A total of 190 nurses and midwives completed a cross-sectional survey. The survey included components on demographics, knowledge of maternal and child healthcare (MCH) and family planning (FP) services, attitude towards family planning services, subjective norms regarding maternal and child healthcare and family planning services, self-efficacy with maternal and child healthcare and family planning services, and intentions to provide maternal and child healthcare and family planning services to adolescents. Pearson’s correlation analysis was conducted to determine the association between knowledge, attitude, subjective norms, self-efficacy, and intention variables for FP and MCH services. A 2-step linear regression analysis was then conducted for both FP and MCH services to predict the intentions to provide FP and MCH services to adolescents.

**Results:**

Self-efficacy to conduct MCH services (β = 0.55, *p* < 0.01) and years of experience as a nurse- midwife (β = 0.14, *p* < 0.05) were associated with stronger intentions to provide the services. Self-efficacy to provide FP services (β = 0.30, *p* < 0.01) was associated with stronger intentions to provide FP services.

**Conclusions:**

Self-efficacy has a strong and positive association with the intentions to provide both MCH and FP services, while there is a moderate association with attitude and norms. There is a need to improve and strengthen nurses’ and midwives’ self-efficacy in conducting both MCH and FP services in order to improve the quality and utilization of the services by adolescents in South Africa.

**Electronic supplementary material:**

The online version of this article (doi:10.1186/s12913-016-1901-9) contains supplementary material, which is available to authorized users.

## Background

The high maternal and neonatal mortality rates in developing countries are a major public health problem, especially in developing countries [1]. Sub-Saharan Africa is among the regions affected by high number of maternal mortality at 201 000 in 2015 [2] and was reported to have contributed to 62% of all maternal deaths worldwide in 2013 [2, 3]. Most of these deaths are a result of preventable causes [3]. Maternal mortality ratio in South Africa (SA) are high at 138 per 100 000 live births [4].

Adolescent mothers are at a much higher risk for maternal mortality [2]. Nearly 16 million girls aged 15 to 19 years give birth every year and about 2.5 million of these births occur to girls aged under 16 years in low- and middle- income countries (LMICs) [4–6]. Pregnancy and childbirth complications are the second cause of death among 15 to 19 year olds globally [2]. Early childbearing increases the risks for maternal and neonatal mortality [5].

In LMICs, infants born to mothers under 20 years of age face a 50% higher risk of being still born or dying in the first few weeks versus those born to mothers aged 20–29 [2, 5]. Newborns born to adolescent mothers are also more likely to have low birth weight, with the risk of long-term effects such as early onset of adult diabetes. In addition, birth outcomes for very young adolescent mothers are particularly poor in terms of increased rates of low birth weights [7, 8]. However, very few studies have investigated the determinants of birth outcomes of pregnant adolescents and adolescent mothers in maternal and child healthcare (MCH) services in SA. Whilst inadequate provision of MCH services to pregnant adolescents and adolescent mothers does not necessarily cause poor birth outcomes, adequate quality provision of these can improve outcomes of both the mother and her newly born infant. Negative attitude of nurses and midwives, lack of respect for the birthing mothers’ views, and, with adolescents particularly, stigmatized sexual activity by the nurses are some of the barriers reported to influence non-utilization of FP and MCH services, such as antenatal care, and contraceptive use in [9]. Lack of youth friendly, dedicated space and youth-friendly trained staff were also reported as barriers to accessing FP services by youth [10]. Fears of being scolded and shouted at, as well as assumptions of being promiscuous by the nurses are specific and serious barriers pertaining to adolescents’ utilization of FP and MCH services [10, 11]. Other reported barriers include the perceived compassion and carrying of traditional birth attendants in comparison to nurses and midwives [9].

Behaviour of healthcare professionals attending to pregnant women and their new-borns is of critical importance for the adequate quality of MCH services: it is one of the factors contributing to non-utilization of MCH services by adolescents [12]. Nurses and midwives’ negative behaviour such as shouting at and scolding clients, is also reported to discourage young people from attending clinics or for follow-up visits [13, 14], although little is known of the underlying determinants. According to the Theory of Planned Behaviour (TPB), an individual’s behaviour as well as the extent to which he or she can control the behaviour is determined by his or her beliefs about having the necessary skills and resources to perform the behaviour [15]. Thus, adopting any behaviour, both desirable and undesirable behaviours, requires the capacity and confidence in one’s ability to attempt the behaviour, that is, self-efficacy or perceived behavioural control. Constructs of TPB have been well documented and researched in their applicability in predicting many behaviours. A meta-analytic review showed that high perceived behavioural control (PBC) and self-efficacy were the strongest predictors of most behaviours, including intentions to perform the behaviours [16]. TBP constructs have also been used to predict behaviours associated with unhealthy sexual and reproductive behaviours, such as poor contraceptive compliance. Condom use among adolescents for example, was associated with high self-efficacy in using the condom [17].

Hence, constructs of TPB might also be associated with healthcare workers’ behaviours related to providing family planning (FP) and MCH services to adolescents. The self-efficacy construct for instance, is an important construct for healthcare providers who believe that they cannot provide certain FP and MCH services adequately. Knowledge and confidence in using partograph, a basic component of managing labour in MCH services, was associated high utilization of partograph among healthcare providers [18–20].

Therefore, the focus of this study will be on identifying the predictors of the intention to provide family planning (FP) and MCH services, as intention is an important predictor of behaviour. In this study, FP services were defined as nurses’ and midwives’ activities and communications to regulate and or prevent conception among adolescents through the practice of contraception or other methods of birth control [2]. MCH services were defined as nurses and or midwives activities and communications given to expectant adolescents and adolescent mothers that is aimed at enhancing their health and that of their unborn baby and the infant [2]. Nurses are all healthcare professional with a nursing qualification, while midwives are nurses who are certified as qualified midwives.

Determining the psychosocial predictors of nurses’ and midwives’ intention to provide MCH services to adolescents will assist in (1) identifying the problems hindering the provision of adequate quality MCH, including FP services to adolescents, (2) providing information to develop and test targeted behaviour change interventions for nurses and midwives in SA, and, ultimately, (3) improving the current maternal and infant health outcomes. Thus, the aim of this study was to determine nurses’ and midwives’ predictors of intention to provide FP and MCH service to adolescents in SA.

## Methods

### Research design

A quantitative cross-sectional study was conducted to gain an understanding of the nurses and midwives intentions to provide FP and MCH services to adolescents. A researcher-developed structured questionnaire was used to obtain information from nurses and midwives in maternal and child healthcare services in the nine provinces in SA. The questionnaire was partly based on constructs derived from Ajzens’ Theory of Planned Behaviour (TPB) [15, 21]. Thus, the questionnaire included questions about attitude, subjective norms, self-efficacy, and intentions to provide FP and MCH services.

### Sampling

The sampling frame consisted of all public health facilities that provide maternity and infant healthcare services in both urban and rural settings of SA. These facilities were stratified into two (2) categories: 1. Public Health Clinics, and 2. Community Day Centres (CDC), Community Health Centers (CHCs) and district hospitals. In this study, CHC is collectively used to group together the CHCs, CDCs and district hospitals as they all conduct secondary level of care compared to clinics which only conducts primary level of care. A representative sample from each stratum was selected using the probability proportional to size approach [22]. The sampling frame consisted of 147 clinics and 34 CDCs, CHCs and district hospitals.

All nine provinces of SA were included in the selection process in order to reflect the diverse demographic and socio-economic profile of the country. Nurses and midwives were recruited at the facility level with the assistance of facility managers. Depending on the size of the facility either all the nurses and midwives or a sample of them were studied. The inclusion criteria for nurses and midwives was: all the nurses whose work included family planning services, caring for pregnant women, delivering babies; and all midwives were eligible to participate in the study. Not all nurses and midwives in these units were available to complete the questionnaire due to the shortage of healthcare professionals in the country, leaving them having to deal with high patient load at the clinics. All nurses and midwives working in MCH and FP units were eligible and recruited to complete the questionnaire. Out of the 250 nurses and midwives that were approached, only 190 agreed to participate, giving a 76% response rate. Work demands and patient overload or the busyness of the clinic were the most common reason for not participating in the study. In other words, the study used a convenience sample of nurses and midwives. The sample size depended on those who were available to participate in the study at the time of data collection in their facility.

### Data collection instruments

Nurses and midwives completed a self-administered questionnaire measuring the determinants of MCH services, and family planning health services behaviour, including demographics as described below. The questionnaire was piloted in a non-study sample and necessary adaptations were made before the study was implemented. The final version of the questionnaire can be found in Additional file 1.

#### Demographic questionnaire

Background information: name of the province, district, location, facility and the type of facility were asked. The gender, participants’ date of birth, position at the facility, the number of years in the position, and education was asked. The variables were coded as following: location (1 = Urban, 2 = Rural), gender (1 = Male, 2 = Female), position at the facility (1 = Registered Nurse, 2 = Registered Midwife, 3 = Staff Nurse, 4 = Enrolled Nurse and Other), type of facility (1 = Clinic, 2 = CHC). Education was (1 = Degree in Nursing) and (2 = Diploma in Nursing and Other). For the purposes of this study, position at facility was grouped into (1 = Registered Midwives, 2 = Registered nurses/Enrolled nurse/Staff nurse, and 3 = other: nurses who were neither registered midwives nor registered nurses).

#### Knowledge of family planning (FP) services

Knowledge of FP services is a 13-item subscale which assesses nurses and midwives’ knowledge of FP services in SA. The statements for the measurement of knowledge of FP services were extracted from the National Contraception Clinical Guidelines: A companion to the National Contraception and Fertility Planning Policy and Service Delivery Guidelines [23]. The statements were provided in the questionnaire where the nurse-midwife was required to confirm whether the statement was true, false or they don’t know. Sample statements are: 1. Combined oral contraceptive pills should be taken at the same time each day, 2. In SA, any female can get an abortion by simply requesting with no reasons given if she is less than 13 weeks pregnant. Scoring options were coded as (1 = True), (2 = False), and (3 = I don’t know) and the total score was calculated based on the number of items answered correctly.

#### Knowledge of maternal and child healthcare (MCH) services

Knowledge of MCH services is an 18-item subscale which assesses nurses and midwives’ knowledge of MCH services in SA. Knowledge of MCH services questions were extracted from the Guidelines for Maternity Care in South Africa (a confidential draft document then), which is now currently the official manual for clinics, CHC and district hospitals in SA [24]. Similar to the knowledge of family planning services 18 statements were provided to the nurses and midwives and were asked to confirm whether the statement was true, false, or they don’t know. Sample items were: 1. Family planning services are a basic component of MCH services, 2. Tetanus immunization is not compulsory for a pregnant woman in SA. Scoring options were coded as (1 = True), (2 = False), and (3 = I don’t know) and the total score was calculated based on the number of items answered correctly.

#### Attitudes towards providing FP services

Attitude toward providing FP services is a ten-item subscale which assesses nurses and midwives’ attitudes towards providing FP services to adolescents in SA. Nurse and Midwives were asked how important it is to them to discuss certain family planning topics with the adolescents in their facility. Sample items were: 1. How important is it to you to discuss injectable contraceptives with adolescents? 2. How important is it to you to discuss termination of pregnancy (TOP) and choices on TOP with adolescents? A Likert scale of 1–5 was used to assess the importance of discussing the topics, where (1 = Very unimportant, 2 = Unimportant, 3 = Neutral, 4 = Important, 5 = Very important).

#### Subjective norms towards providing FP and MCH services

Subjective norms subscale is a six-item (for FP) and 5-item (for MCH) subscale which assesses general norms of the nurses and midwives’ colleagues at the facility they work in. Nurses and Midwives were asked how strongly they agreed with certain FP and MCH statements regarding their colleagues’ views. The items were 6 for FP and 5 for MCH services. A Likert scale was also used to assess their subjective norms for FP and MCH separately. Sample items were: 1. Most of your colleagues think that you should provide female condoms to adolescent girls (for FP services), 2. Most of your colleagues think that you should discuss delivery options with pregnant adolescent girls (for MCH services). Scoring options were 1 = Strongly disagree, 2 = Disagree, 3 = Neutral, 4 = Agree, 5 = Strongly agree).

#### Self-efficacy to provide FP and MCH services

Self-efficacy is one’s belief in his or her capabilities to perform a recommended behaviour [25]. In this study, self-efficacy is defined as the nurses’ and midwives’ beliefs and confidence in themselves performing a recommended FP and MCH services for adolescents in SA. Nurses and Midwives were asked to indicate how confident they were to provide FP services and MCH services. The total items were 7 for FP and 10 for MCH services. To measure FP self-efficacy a sample question was: how confident are you that you will be able to provide injectable contraceptives to adolescent girls? To measure MCH self-efficacy a sample question was: how confident are that you will be able to provide immunization for diphtheria, tetanus, and pertussis to pregnant adolescents. Scoring options were 1 = Not confident at all, 2 = Not confident, 3 = Unsure, 4 = Confident, 5 = Very confident.

#### Intention to provide FP and MCH services

Intention is a 16-item (for FP services) and ten-item (for MCH services) which assesses nurses and midwives’ intentions to provide family planning services to adolescents in SA. Nurses and Midwives were asked to indicate whether they intended to provide certain FP and MCH services. Sample items were: the next time an adolescent girl needs TOP, I intend to provide (for FP services), and the next time a pregnant adolescent needs advice on delivery options, I intend to provide the advice (for MCH services). The total number of items was 16 for FP services and 10 for MCH services. The Likert scale scoring options were 1 = Definitely not, 2 = Not, 3 = Unsure, 4 = Yes, 5 = Definitely yes.

### Data analysis

Data were analyzed using SPSS version 21 [26]. Descriptive statistics were conducted to get an overview of the sample characteristics. Mean scores were also calculated in order to gain more information about the nurse- midwives’ personal determinants regarding adolescents’ MCH and FP services. Pearson’s correlation analysis was then conducted to determine the association between knowledge, attitude, subjective norms, self-efficacy, and intention variables for FP and MCH services. The strengths for Pearson’s correlation were classified as weak (0.10 ≤ *r* < 0.30), moderate (0.30 ≤ *r* < 0.50) or strong (*r* ≥ 0.50) [27]. All variables, including the demographics were then taken into multiple linear regression analysis to predict the intentions to provide FP and MCH services to adolescents by the nurses and midwives in this study. Intentions to provide FP and MCH services are the outcome variables for the linear regression analysis.

Prior to the linear regression analysis, exploratory factor analysis was conducted to assess the items making up the scales in order to group together items that were closely related [28]. Thirteen items from the knowledge of FP services, and 18 items from the knowledge of MCH services were put through factor analysis in SPSS. Kaiser-Mayer-Oklin (KMO) test for the FP knowledge subscale met the recommended value at 0.6 with the Bartlett’s test reaching the significance level, *p* < 0.01, and the KMO value for MCH knowledge subscale was more than the recommended 0.6 with a significant Bartlett’s test, *p* < 0.01 [28]. Ten items of FP attitudes, 6 items on subjective norms for FP and 5 items for MCH, 7 items self-efficacy for FP and MCH services, and 16 items on intentions to provide FP and MCH services were also put through the factor analysis. All these subscales were above the minimum recommended 0.6 value of the KMO with a significant Bartlett’s test *p* < 0.01 [28]. Kaiser-Mayer-Oklin and Bartlett’s tests were used to assess the suitability of the data for the planned analysis to ascertain that no assumptions were violated, as well as the appropriateness of the factor analysis that was used to assess the items making up the scales used [28]. The KMO values confirmed that the variables used in this study were fit for further analysis, which the study intended to conduct.

A 2-step linear regression analysis was conducted for both FP and MCH services. In step 1, only demographics: Location (urban or rural), position of the staff, education level, and years of experience were added into the FP and MCH regression models. In step 2, demographics and the psychosocial variables: FP knowledge, FP attitude, FP norms, and FP self-efficacy were added into the FP model. MCH psychosocial variables: MCH knowledge, MCH norms, and MCH self-efficacy were added into the MCH model.

Collinearity statistics showed that the variables entered into the linear regression model did not violate the multi-collinearity assumptions. All variables for both FP and MCH services obtained an acceptable variance inflation factor (VIF) that is below 10, the cut off value [28, 29]. Only variables with a p- value of less than 0.05 (*p* < 0.05) were considered significant variables in this study.

## Results

### Sample characteristics

The total sample consisted of 190 nurses and midwives from the nine (9) provinces of SA. Only ten (5.2%) of the nurses and midwives were male, the majority was female 183 (94.8%). The mean age of the nurses and midwives was 46.0 (SD = 10.0), with 34 (21.4%) being 30–39 years of age; 46 (28.9%) being 40–49 years of age; and 52 (32.7%) being 50–59 years old. Majority of the nurses and midwives, 129 (73.3%) worked in a rural setting. The Public Health Clinic was the common type of facility many worked in (81.2%), while only 18.8% worked in CHCs. Nearly a quarter of them (25.3%) had a degree in nursing and the majority (74.7%) had a diploma in nursing or other levels of education. Further sample characteristics are described below in Table 1.

**Table 1:** Demographic characteristics of the health care workers (*n* = 190)

Total		Count (*n*)	Percent (%)
		190	100
Gender	Male	9	4.8
	Female	179	95.2
Age group	20–29	12	7.8
	30–39	32	20.8
	40–49	45	29.2
	50–59	51	33.1
	≥ 60	14	9.1
Province	Gauteng	12	6.6
	Mpumalanga	16	8.8
	Western Cape	21	11.6
	Eastern Cape	23	12.7
	Northern Cape	27	14.9
	Free State	24	13.3
	Kwazulu-Natal	17	9.4
	Limpopo	26	14.4
	North-West	15	8.3
Type of facility where the nurses and midwives work	Clinic	147	81.2
	CHC	34	18.8
Facility Setting where the nurses and midwives work	Urban	53	28.3
	Rural	134	71.7
Current position of the nurses and midwives at the facility where they work	Registered Midwife	57	30.0
	Registered Nurse/Staff Nurse/Enrolled Nurse	104	54.7
	Other: nurses who are neither registered midwives or nurses	29	15.3
Years of experience	1–5 years	49	28.0
	6–10 years	46	26.3
	11–15 years	27	15.4
	16–20 years	15	8.6
	21–30 years	34	19.4
	≥ 31 years	4	2.3
Educational background	Degree in Nursing	48	27.0
	Diploma in Nursing and Other: nurses with less than a degree or diploma qualification	130	73.7

### Mean knowledge scores for FP and MCH services

The mean knowledge score of FP services for all nurses and midwives was 9.6 (SD = 1.7), where 25.6% scored 5–8 (lowest scores), 61.9% scored 9–11 (median scores) and 12.5% scored 12–13 (highest scores). With regards to MCH services, the mean knowledge score among nurses and midwives in the study was 16.2 (SD = 1.9). Nurses and midwives had moderately high knowledge of MCH practices with 55.3% achieving a mean score of 17–18 (the highest scores), and 38.8% achieving a mean score of 13–16 (median scores).

### Correlations between nurses and midwives’ psychosocial determinants and intentions to provide FP and MCH services

The relationship between nurses and midwives’ psychosocial determinants and intentions to provide FP and MCH services were investigated. Table 2 shows the results of Pearson’s correlation analysis. No significant correlations were found between FP knowledge, MCH knowledge and the intentions to provide FP and MCH services to adolescents. Positive correlation was observed for FP attitudes (*r* = 0.29, *p* < 0.01), and FP norms (*r* = 0.27, *p* < 0.01). The moderate correlations between FP attitude and FP norms, show that a more positive FP attitude, better FP norms, are more likely to increase the nurse- midwives’ intentions to provide FP services to adolescents in SA. There was a strong correlation between FP self-efficacy and intentions to provide FP services (*r* = 0.44, *p* < 0.01). This positive correlation suggest that the more confident the nurse- midwives are in providing FP services to adolescents the higher the intentions to provide the FP services to adolescents. A moderate positive correlation between MCH norms and intentions to provide MCH was also observed (*r* = 0.27, *p* < 0.01). MCH self-efficacy correlation with intentions to provide MCH was also a positive correlation (*r* = 0.58, *p* < 0.01). MCH self-efficacy had a fairly strong correlation indicating that the more confident the nurse or midwife is (self-efficacy) with conducting MCH services to adolescents the more likely that he or she intends to provide MCH services to adolescents.

**Table 2:** Pearson correlations between psychosocial determinants and intentions to provide family planning (FP) services and mother and child healthcare (MCH) services

Psychosocial predictors	FP Intention r (95% CI)	MCH Intention r (95% CI)
FP knowledge	−0.12 (95% CI: −0.28–0.05)	
FP attitudes	0.29** (95% CI: 0.10–0.46)	
FP norms	0.27** (95% CI: 0.12- 0.42)	
FP self-efficacy	0.44** (95% CI: 0.29–0.56)	
MCH services knowledge		−0.05 (95% CI: −0.21–0.11)
MCH norms		0.27** (95% CI: 0.13–0.41)
MCH self-efficacy		0.58** (95% CI: 0.47–0.67)

**Correlation is significant at *p* <0.01

### Multivariate predictors of nurse- midwives intentions to provide FP and MCH services to adolescents

A hierarchical linear regression analysis was conducted in Tables 3 and 4 below. Table 3 Model: in step 1, demographic characteristics we fed into the regression model to determine the variables predicting intentions to provide FP services among nurses and midwives. None of variables were significant in this step. In step 2 all variables were fed into the regression model including those in step 1. None of the variables that were included in step 1 were significant in step 2. One variable in step 2, FP Self-efficacy variable was significant, with p <0.01 (see Table 3). FP self-efficacy (β = 0.30, *p* < 0.01) was the only significant positive predictor of intentions to provide FP services. This suggests that a stronger confidence in conducting FP services to adolescents is correlated with stronger intentions to provide FP services.

**Table 3:** Regression analysis predicting intentions to conduct FP services among nurses and midwives

	*B*	*SE b*	β	*P*-value	95% CI	Variance Inflation Factor (VIF)
Step 1						
Constant	41.88					
Location	−0.90	0.91	−0.11	0.32	−2.71–0.89	1.08
Staff position	0.16	0.28	0.06	0.57	−0.39–0.73	1.07
Staff education	0.86	0.93	0.10	0.36	−0.98–2.72	1.06
Years of experience	−0.07	0.26	−0.03	0.79	−0.58–0.45	1.04
Step 2						
Constant	27.23					
Location	−1.36	0.82	−0.16	0.10	−2.98–0.27	1.13
Staff position	0.18	0.25	0.07	0.47	−0.32–0.68	1.08
Staff education	0.97	0.83	0.11	0.24	−0.68–2.63	1.08
Years of experience	0.16	0.24	0.06	0.50	−0.31–0.63	1.11
FP Knowledge	−0.33	0.21	−0.15	0.12	−0.74–0.08	1.04
FP Attitude	0.23	0.12	0.19	0.06	−0.01–0.47	1.21
FP Norms	0.17	0.12	0.15	0.16	−0.07–0.42	1.28
FP Self-efficacy	0.49	0.18	0.30**	< 0.001	0.14–0.85	1.38

Step 1: Δ *R* ^2^ = 0.021; Δ *F* = 0.47; *p* > 0.05

Step 2: Δ *R* ^2^ = 0.25; Δ *F* = 7.32; *p* < 0.01

**p < 0.01

**Table 4:** Regression analysis predicting intention to conduct MCH services among nurses and midwives

	*B*	*SE b*	β	*P*-value	95% CI	Variance Inflation Factor (VIF)
Step 1						
Constant	46.82					
Location	0.06	0.55	0.01	0.91	−1.03–1.15	1.08
Staff position	0.11	0.17	0.05	0.52	−0.23–0.45	1.07
Staff education	0.17	0.57	0.03	0.76	−0.94–1.29	1.06
Years of experience	0.31	0.16	0.16	0.05	−0.01–0.62	1.04
Step 2						
Constant	19.45					
Location	−0.24	0.45	−0.04	0.60	−1.14–0.66	1.12
Staff position	0.06	0.14	0.03	0.66	−0.22–0.34	1.09
Staff education	0.33	0.46	0.05	0.48	−0.58–1.24	1.07
Years of experience	0.27	0.13	0.14*	0.04	0.01–0.52	1.07
MCH Knowledge	0.01	0.10	0.01	0.95	−0.19–0.21	1.05
MCH Norms	0.15	0.09	0.11	0.11	−0.04–0.33	1.15
MCH Self-efficacy	0.51	0.06	0.55**	< 0.001	0.38–0.63	1.12

Step 1: Δ *R* ^2^ = 0.26; Δ *F* = 0.99; *p* > 0.05

Step 2: Δ *R* ^2^ = 0.35; Δ *F* = 26.85; *p* < 0.01

*p < 0.05

**p < 0.01

With regards to MCH services, Table 4 Model: the same process was followed for model predicting intentions to provide MCH services among the nurses and midwives. Again, none of the variables in step 1 were significant. However, years of experience (β = 0.14, *p* < 0.05) and a stronger confidence in conducting MCH services- MCH self-efficacy (β = 0.55, *p* < 0.01) were correlated with providing MCH services to adolescents (see Table 4).

Considering the Benferroni adjustment where *p* ≤ 0.0071 (i.e., 0.05/7) is significant, both FP self-efficacy and MCH self-efficacy remain significant predictors of intentions to provide FP services and MCH services, respectively.

## Discussion

This study examined predictors of nurses and midwives intentions to provide family planning and maternal and child healthcare services to adolescents in SA. Self-efficacy in conducting MCH services is the strongest positive predictor of intentions to provide MCH services to adolescents in SA. Likewise, self-efficacy in providing FP services is the strongest predictor of intentions to provide such services to adolescents. This highlights the significance of nurses’ and midwives’ confidence (self-efficacy) in family planning and maternal and child healthcare best practice, particularly towards adolescent FP and MCH needs. If the nurses and midwives in MCH services perceive themselves as incapable to provide FP and MCH best practice, the quality of their service should not be expected to be adequate.

These findings also support previous research where it is well documented that nurses and midwives typically do not feel comfortable to provide FP services, such as contraceptives to adolescents for various reasons including fear of encouraging their sexual activities [11, 30, 31]. Insufficient training regarding adolescent’s SRH issues is among the reasons nurses and midwives are reluctant to provide FP services to adolescents [31–35]. If nurses and midwives in MCH services in SA can be adequately skilled to attend to adolescents SRH needs, they most likely would feel confident to provide FP services to adolescents and thus increases the intentions to provide the services. This in turn, can have a positive effect on adolescents’ SRH and can potentially increase the use of FP services by adolescents. Increased use of FP services by adolescents is very important in reducing the high teenage pregnancy rates in SA [36].

These findings may also explain the poor quality of care in maternal and child healthcare facilities. Poor quality of care is now regarded as a barrier for antenatal and postnatal care, especially in low- and middle- income countries (LMICs) [37–39]. Evidently, adequate utilization of FP and MCH services, particularly by adolescents relies on good quality services. A number of studies in sub-Saharan Africa reported poor quality of care at delivery [37, 40, 41]. This is concerning as good quality best practices are necessary in MCH services in order to reduce maternal and child morbidity and mortality rates. Additionally, this exacerbates the situation pertaining to adolescents’ sexual and reproductive healthcare (SRH) service needs as adolescents’ access to and utilization of MCH services is already poor [13–15].

Nurses and midwives’ FP attitudes and norms are also positively related to intentions to provide FP services to adolescents. This is in line with previous research showing that nurses and midwives attitudes towards adolescents’ use of contraceptives or abortion services for example, are unfavourable [9, 30, 42, 43]. Moreover, nurses and midwives feel if they provide adolescents with contraceptives, they are encouraging adolescent’s sexual activity [11]. Clearly, the thoughts about adolescents’ sexual activity being immoral affects some nurses’ clinical practices, such as providing adolescents with contraceptives. These negative attitudes and persistent norms towards adolescents’ sexual activities and consequently FP use and or needs pose a serious challenge when it comes to improving contraceptive use among adolescents and alleviate teenage pregnancy in SA. Contraceptive use among adolescents in SA is reported to be fairly poor [11, 36], and this finding indicates that nurses and midwives attitudes towards, and norms related to adolescents’ FP services maybe associated. Efforts to increase use of FP services by adolescents should be prioritize to curb teenage pregnancy, and consequently reduce maternal and child morbidity and mortality rates.

Negative attitude towards abortion among nurses and midwives in many sub-Saharan African countries have also been reported, despite the global agreements on termination of pregnancy as a SRH right for all women and adolescents [44, 45]. This presents a challenge in improving adolescents’ use of FP services, including termination of pregnancy to prevent and or abort unwanted pregnancies. Therefore, the need to modify nurses and midwife attitudes and norms regarding adolescents’ FP services is critical. Moreover, nurses and midwives attitudes towards adolescents’ FP services and abortion, as well FP norms play a key role in determining their intentions to provide the services and therefore require improvement.

An important limitation in interpreting the findings of this study is that despite being based on the TPB [21, 23], the questionnaire has not been used previously. However, the subscale analyses of the questionnaire constructs confirmed the reliability of the quantitative measurement to collect behavioral related determinants among healthcare professionals in MCH services. Further validation of these measurements in future research is needed. There is no information of participants who were not able to participate, other than their profession as nurses or midwives. However, non-participation is most likely not selective due to the study topic as the most import reason mentioned for non-participation was lack of time due to work demands. Despite this limitation, this study brings forth useful information regarding the psychosocial determinants of MCH best practice among nurse- midwives in SA. The cross-sectional nature of this study provides a snapshot of personal determinants of the nurses and midwives in MCH services which require improvement for adolescents FP and MCH utilization. Furthermore, the sample of nurses and midwives from different socio-economic and cultural backgrounds is in line with the general population of nurses and midwives in FP and MCH services in SA.

## Conclusions

Self-efficacy is a strong positive predictor of intentions to provide both FP and MCH services. There is a need to improve and strengthen nurses’ and midwives’ self-efficacy in conducting both MCH and FP services to adolescents in SA, in order to increase intentions to provide these services. High self-efficacy and high intentions to provide FP and MCH services would increase the provision of the services and also likely to increase the utilization of services by adolescents in the country. Interventions aiming at improving self-efficacy should use behaviour change methods such as self re-evaluation and planning coping response methods [46]. This ultimately may lead to improved child health outcomes and reduction of the burden of maternal and child morbidity and mortality, as well as increased family planning utilization by adolescents in SA. The recruitment process of nurses and midwives providing SRH services also needs to be revised to further strengthen and improve adolescents’ health outcomes in SA.

## Additional file

Additional file 1: Questionnaire. (PDF 382 kb)

## Abbreviations

CDC: Community day center
CHC: Community health center
FP: Family planning
HSRC: Human Sciences Research Council
KMO: Kaiser-Mayer-Oklin
LMIC: Low- and middle- income country
MCH: Maternal and child healthcare
SA: South Africa
SPSS: Statistical Package for the Social Sciences
SRH: Sexual and reproductive healthcare
TPB: Theory of planned behaviour
VIF: Variance inflation factor
