# Supplementary material for: Predictors of nurses’ and midwives’ intentions to provide maternal and child healthcare services to adolescents in South Africa
Source: BMC Health Serv Res. 2016 Nov 15;16:658. doi: 10.1186/s12913-016-1901-9 (PMC5111177; doi:10.1186/s12913-016-1901-9)
Supplement: Additional file 1: — Questionnaire. (PDF 382 kb) [file 12913_2016_1901_MOESM1_ESM.pdf]

KNOWLEDGE, ATTITUDES AND BEHAVIOUR OF HEALTH WORKERS WORKING IN MATERNAL AND CHILD HEALTHCARE SERVICES

A: FACILITY BACKGROUND INFORMATION				
**No.**	**Question & Filters**	**Coding Categories**		**Code**
1	Province name			
2.	District name			
3	Location:	Urban		
		Rural		
4	Facility Name:			
5	Type of facility	Clinic	1	
		Community Day Centre	2	
		Community Health Centre	3	
		Hospital	4	
6	Date of Interview:			
7	Name of the Interviewer:			

B: PARTICIPANT BACKGROUND INFORMATION				
**No.**	**Question & Filters**	**Coding Categories**		**Code**
1	Gender of the participant:	Male		
		Female		
2	Participant date of birth:<br><br>DD/MM/YYYY			
3	What is your position at this facility?	Registered Nurse		
		Registered Midwife		
		Staff Nurse		
		Enrolled Nurse		
		Other (specify)		
4	How long have you been working in this position?			
5	What is your employment status at this	Full time		

	current job?	Part-time	
		Contract	
		Other (specify)	
6	What is your educational background?	Diploma in Nursing	
		Degree in Nursing	
		Other (specify)	
7	In the last twelve months have you received any antenatal care Continuing Professional Development (CPD) training on the following?	Yes	No
	a) Principles of antenatal care	1	2
	b) First antenatal visit	1	2
	c) Subsequent antenatal visits	1	2
	d) BANC checklist	1	2
8	In the last twelve months have you received any labour and puerperium CPD training on the following?	Yes	No
	a) Normal labour and puerperium	1	2
	b) Abnormalities of first stage of labour	1	2
	c) Abnormalities of second stage of labour	1	2
	d) Caesarean section	1	2
	e) Emergencies during labour	1	2
	f) Immediate care of the newborn	1	2
	g) Abnormalities of the third and fourth stages of labour	1	2
	i) Abnormalities of the puerperium	1	2
9	In the last twelve months have you received any Anaesthesia and resuscitation CPD training on the following?	Yes	No
	a) Pre-anaesthetic evaluation	1	2
	b) Anaesthesia for caesarean section	1	2
	c) Complications during obstetric anaesthesia	1	2
	d) Cardio-pulmonary resuscitation in pregnancy	1	2
10	In the last twelve months have you received any hypertensive disorders of pregnancy CPD training on the following?	Yes	No
	a) Hypertension in pregnancy	1	2
	b) Classification and grading	1	2
	c) Pathophysiology of pre-eclampsia	1	2
	d) Management of pre-eclampsia	1	2
	e) Labour, delivery and postpartum care	1	2
	f) Chronic hypertension	1	2
11	In the last twelve months have you received any pregnancy problems CPD training on the following?	Yes	No
	a) Intrauterine growth restriction	1	2

	b) Intrauterine death	1	2
	c) Antepartum haemorrhage	1	2
	d) Multiple pregnancy	1	2
	e) Breech presentation and transverse lie	1	2
	f) Preterm labour	1	2
	g) Premature rupture of the membranes	1	2
	h) Chorioamnionitis	1	2
	i) Post term pregnancy	1	2
	j) Induction of labour with a live baby	1	2
	k) Previous caesarean section	1	2
	l) Rhesus incompatibility	1	2
	m) Poor obstetric history	1	2
	n) Birth defects and genetic disorder	1	2
12	In the last twelve months have you received any medical disorders in pregnancy CPD training on the following?	Yes	No
	a) Anaemia	1	2
	b) Diabetes mellitus	1	2
	c) Cardiac diseases	1	2
	d) Asthma	1	2
	e) Thromboembolism	1	2
	f) Epilepsy	1	2
13	In the last twelve months have you received any medical disorders in pregnancy CPD training on the following?	Yes	No
	a) Abnormal vaginal discharge	1	2
	b) Genital ulcers	1	2
	c) Genital warts	1	2
	d) Syphilis	1	2
	e) Malaria	1	2
	f) Urinary tract infections	1	2
	g) Pneumonia and tuberculosis	1	2
	h) Human immunodeficiency virus	1	2
	i) Prevention of mother to child transmission	1	2
14	In the last twelve months have you received any prevention of mother to child transmission and management of HIV positive CPD training on the following?	Yes	No
	a) Risk factors for Mother-to-child HIV Transmission	1	2
	b) Antenatal Care	1	2
	c) Management of Labour	1	2
	d) Post- Delivery care	1	2
	e) Termination of Pregnancy	1	2
	f) Other Considerations	1	2
15	In the last twelve months have you received any training on Integrated Management of Pregnancy and Childbirth (IMPAC)?	Yes	No

16	In the last twelve months have you received any training on Integrated Management of Childhood Illness (IMCI)?	Yes		No		
17	In the last twelve months have you received any training on Growth Monitoring?	Yes		No		
18	In the last twelve months have you received any training regarding the Implementation of the DoH Guidelines on Child and Maternal Services?	Yes		No		
**C. REPRODUCTIVE HEALTH SERVICES: FAMILY PLANNING BEHAVIOUR**						
**C1. PROVISION OF FAMILY PLANNING SERVICES**						
	During the past month (30 days), how often did you provide the following family planning services?<br>*Never (0 days=1), Rarely (1-2 days=2), Sometimes (3-9 days=3), Often (10-19 days= 4) and Very often (>20=5)*					
**No.**	**Question & Filters**					
1.	Do you provide termination of pregnancy (TOP) services in this facility? YES/NO ……<br>**If no skip to no2**<br><br>Termination of pregnancy to:<br>a) teenage females	Never (1)	Rarely (2)	Sometimes (3)	Often (4)	Very often (5)
	b) unmarried persons	1	2	3	4	5
	c) married persons	1	2	3	4	5
2.	Combined oral contraceptive pills to:<br>a) Teenage females	1	2	3	4	5
	b) unmarried persons	1	2	3	4	5
	c) to married persons	1	2	3	4	5
3.	Progestin only contraceptive pills to:<br>a) Teenage females	1	2	3	4	5
	b) to unmarried persons	1	2	3	4	5
	c) to married persons	1	2	3	4	5
4.	Combined injectable contraceptives to:<br>a) Females	1	2	3	4	5
	b) to unmarried persons	1	2	3	4	5
	c) to married persons	1	2	3	4	5
5.	Progestin only injectable contraceptives to:<br>a) teenage females	1	2	3	4	5
	b) unmarried persons	1	2	3	4	5
	c) married persons	1	2	3	4	5
6.	Male condoms to:<br>a) Females	1	2	3	4	5
	b) unmarried persons	1	2	3	4	5

	c) married persons	1	2	3	4	5
7.	Female condoms to:<br>a) teenage females	1	2	3	4	5
	b) unmarried persons	1	2	3	4	5
	c) married persons	1	2	3	4	5
8.	Intrauterine contraceptive devices to: **CHC only**<br>a) Teenage females	1	2	3	4	5
	b) unmarried persons	1	2	3	4	5
	c) married persons	1	2	3	4	5
9.	Implants to:<br>**CHC only**<br>a) teenage females	1	2	3	4	5
	b) unmarried persons	1	2	3	4	5
	c) married persons	1	2	3	4	5
10.	Cycle beads for standard days method to:<br>a) teenage females	1	2	3	4	5
	b) unmarried persons	1	2	3	4	5
	c) married persons	1	2	3	4	5
11.	Emergency contraceptive pills to:<br>a) teenage females	1	2	3	4	5
	b) unmarried persons	1	2	3	4	5
	c) married persons	1	2	3	4	5
12.	Male sterilisation to:<br>**CHC only**<br><br>a) teenage males	1	2	3	4	5
	b) unmarried persons	1	2	3	4	5
	c) married persons	1	2	3	4	5
13.	Female sterilisation to:<br>**CHC only**<br>a) teenage females	1	2	3	4	5
	b) unmarried persons	1	2	3	4	5
	c) married persons	1	2	3	4	5

C2. KNOWLEDGE OF FAMILY PLANNING

The following statements are about family planning knowledge. Please tell me if you think the statement is true (1), false (2,) or if you do not know (3).

		True	False	I don't know
1)	In South Africa, any female of any age can get an abortion by simply requesting with no reasons given, if she is less than 13 weeks pregnant.	1	2	3
2)	In South Africa, if a female is aged 12, and is 20 weeks pregnant, she can get an abortion if her own physical or mental health is at stake.	1	2	3
3)	In South Africa, if a female is aged 12, and is 20 weeks pregnant, she can get an abortion if the baby will have severe mental or physical abnormalities.	1	2	3

4)	In South Africa, if a female is aged 12, and is 20 weeks pregnant, she can get an abortion if she is pregnant as a result of incest.	1	2	3
5)	In South Africa, if a female is aged 12, and is 20 weeks pregnant, she can get abortion if she is pregnant as a result of rape.	1	2	3
6)	In South Africa, if a female is more than 20 weeks pregnant, she can get an abortion only if her life or the life of her foetus is in danger.	1	2	3
7)	Combined oral contraceptive pills should be taken at the same time each day.	1	2	3
8)	Combined injectable contraceptives can be given to adult females.	1	2	3
9)	Combined injectable contraceptives can be given to teenage females.	1	2	3
10)	Cycle beads for standard days methods can be provided to females.	1	2	3
11)	Emergency contraceptive pills can be provided to teenage females.	1	2	3
12)	Only women 40 years and above require regular pap smears.	1	2	3
13)	HPV screening should be done on pregnant women only.	1	2	3

C3. ATTITUDES TOWARDS FAMILY PLANNING

How important is it to you to discuss the following with patients?
Very unimportant (1), Unimportant (2), Neutral (3), Important (4), Very important (5)

		Very unimportant (1)	Unimportant (2)	Neutral (3)	Important (4)	Very Important (5)
1.	Termination of pregnancy and choices on TOP with:<br>a) teenage females	1	2	3	4	5
	b) unmarried persons	1	2	3	4	5
	c) married persons	1	2	3	4	5
2.	Oral contraceptive pills with:<br>a) teenage females	1	2	3	4	5
	b) unmarried persons	1	2	3	4	5
	c) married persons	1	2	3	4	5
3.	Injectable contraceptives with:<br>a) teenage females	1	2	3	4	5
	b) unmarried persons	1	2	3	4	5
4.	Female condoms with:<br>a) teenage females	1	2	3	4	5
	b) unmarried persons	1	2	3	4	5
	c) married persons	1	2	3	4	5
5.	Intrauterine contraceptive devices with:<br>**CHC Only**<br>a) teenage females	1	2	3	4	5
	b) unmarried persons	1	2	3	4	5

	c) married persons	1	2	3	4	5
6.	Implants with:<br>**CHC Only**<br>a) teenage females	1	2	3	4	5
	b) unmarried persons	1	2	3	4	5
	c) married persons	1	2	3	4	5
7.	Cycle beads for standard days method with:<br>a) teenage females	1	2	3	4	5
	b) unmarried persons	1	2	3	4	5
	c) married persons	1	2	3	4	5
8.	Emergency contraceptive pills with:<br>a) teenage females	1	2	3	4	5
	b) unmarried persons	1	2	3	4	5
	c) married persons	1	2	3	4	5
9.	Male sterilisation with:<br>**CHC Only**<br>a) teenage males	1	2	3	4	5
	b) unmarried persons	1	2	3	4	5
	c) married persons	1	2	3	4	5
10.	Female sterilisation with:<br>**CHC & Only**<br>a) teenage females	1	2	3	4	5
	b) unmarried persons	1	2	3	4	5
	c) married persons	1	2	3	4	5

C4. SUBJECTIVE NORMS TOWARDS FAMILY PLANNING* these are norms among colleagues

**How strongly do you agree with the following statements?**		Strongly Disagree	Disagree	Neutral	Agree	Strongly Agree
1)	Most of your colleagues think that you should provide termination of pregnancy to pregnant teenage females.<br>**CHC Only**	1	2	3	4	5
2)	Most of your colleagues think that you should provide oral contraceptive pills to:<br>a) teenage females	1	2	3	4	5
	b) unmarried persons only	1	2	3	4	5
3)	Most of your colleagues think that you should provide injectable contraceptives to: a) teenage females	1	2	3	4	5
	b) unmarried persons only	1	2	3	4	5
4)	Most of your colleagues think that you should provide female condoms to:<br>a) teenage females	1	2	3	4	5
	b) unmarried persons only	1	2	3	4	5
5)	Most of your colleagues think that you	1	2	3	4	5

	should provide intrauterine contraceptive device to: (**CHC Only)**<br><br>a) teenage females					
	b) unmarried persons only	1	2	3	4	5
6)	Most of your colleagues think that you should provide emergency contraceptive pills to:<br>a) teenage females	1	2	3	4	5
	b) unmarried persons	1	2	3	4	5
	c) married persons	1	2	3	4	5

**C5. SELF-EFFICACY TO PROVIDE FAMILY PLANNING**						
**Please indicate how confident you are to provide the following services:**		Not confident at all	Not confident	Unsure	Confiden t	Very confident
1.	How confident are you that you will be able to prepare termination of pregnancy for: (**CHC Only)**<br>a) teenage females	1	2	3	4	5
	b) unmarried women	1	2	3	4	5
	c) married women	1	2	3	4	5
2.	How confident are you that you will be able to provide oral contraceptive pills to:<br>a) teenage females	1	2	3	4	5
	b) unmarried persons	1	2	3	4	5
	c) married persons	1	2	3	4	5
3.	How confident are you that you will be able to provide injectable contraceptives to:<br>a) teenage females	1	2	3	4	5
	b) unmarried persons	1	2	3	4	5
	c) married persons	1	2	3	4	5
4.	How confident are you that you will be able to provide female condoms to:<br>a) teenage females	1	2	3	4	5
	b) unmarried persons	1	2	3	4	5
	c) married persons	1	2	3	4	5
5.	How confident are you that you will be able to provide intrauterine contraceptive device to:<br>(**CHC Only)**<br><br>a) teenage females	1	2	3	4	5
	b) unmarried persons	1	2	3	4	5
	c) married persons	1	2	3	4	5
6.	How confident are you that you will be	1	2	3	4	5

	able to provide implants to:<br>(**CHC Only)**<br>a) teenage females					
	b) unmarried persons	1	2	3	4	5
	c) married persons	1	2	3	4	5
7.	How confident are you that you will be able to provide emergency contraceptive pills to:<br>a) teenage females	1	2	3	4	5
	b) unmarried persons	1	2	3	4	5
	c) married persons	1	2	3	4	5

C6. INTENTION TO PROVIDE FAMILY PLANNING

**Please indicate whether you intend to provide the following family planning services**		Definitely not	Not	Unsure	Yes	Definitely yes
1)	The next time a pregnant teenager needs termination of pregnancy, I intend to do it.<br>(**CHC Only)**	1	2	3	4	5
2)	The next time a teenage female needs oral contraceptive pills, I intend to provide them.	1	2	3	4	5
3)	The next time a married woman needs oral contraceptive pills, I intend to provide them.	1	2	3	4	5
4)	The next time an unmarried woman needs oral contraceptive pills, I intend to provide them.	1	2	3	4	5
5)	The next time a teenage female needs injectable contraceptives, I intend to provide it.	1	2	3	4	5
6)	The next time a married woman needs injectable contraceptives, I intend to provide it.	1	2	3	4	5
7)	The next time an unmarried woman needs injectable contraceptives, I intend to provide it.	1	2	3	4	5
8)	The next time a teenage female needs an intrauterine contraceptive device, I intend to provide it.<br>(**CHC Only)**	1	2	3	4	5
9)	The next time a married woman needs an intrauterine contraceptive device, I intend to provide it.<br>(**CHC Only)**	1	2	3	4	5
10)	The next time an unmarried woman needs an intrauterine contraceptive device, I intend to provide it.<br>(**CHC Only)**	1	2	3	4	5

11)	The next time a teenage female needs an implant, I intend to provide it. (**CHC Only)**	1	2	3	4	5
12)	The next time a married woman needs an implant, I intend to provide it. (**CHC Only)**	1	2	3	4	5
13)	The next time an unmarried woman needs an implant, I intend to provide it. (**CHC Only)**	1	2	3	4	5
14)	The next time a teenage female needs emergency contraceptive pills, I intend to provide it.	1	2	3	4	5
15)	The next time a married woman needs emergency contraceptive pills, I intend to provide it.	1	2	3	4	5
16)	The next time an unmarried woman needs emergency contraceptive pills, I intend to provide it.	1	2	3	4	5

D: MATERNAL AND CHILD CARE SERVICES

**D1. PROVISION OF MATERNAL CARE SERVICES**						
	During the past month (30 days), how often did you provide the following maternal care services? Never or 0 days=1, Rarely or 1-2 days= 2, Sometimes or 3-9=3, Often 10-19 days= 4 and Very often >20 days= 5					
1.	Routine prenatal physical examination (height, weight, BP, fetal heart sounds, size and position of the fetus) among:<br>a) teenage females	Never (1)	Rarely (2)	Sometimes (3)	Often (4)	Very often (5)
	b) unmarried women	1	2	3	4	5
	c) married women	1	2	3	4	5
2.	Routine prenatal blood screening (CBC, HepB, HIV, syphilis, diabetes) among:<br>a) teenage females	1	2	3	4	5
	b) unmarried women	1	2	3	4	5
	c) married women	1	2	3	4	5
3.	Provide iron supplements to:<br>a) Teenage females	1	2	3	4	5
	b) unmarried women	1	2	3	4	5
	c) married women	1	2	3	4	5
4.	Provide folate supplements to:<br>a) teenage females	1	2	3	4	5
	b) unmarried women	1	2	3	4	5
	c) married women	1	2	3	4	5
5.	Booster immunization for diphtheria, tetanus and pertussis to:<br>a) teenage females	1	2	3	4	5

	b) unmarried women	1	2	3	4	5
	c) married women	1	2	3	4	5
6.	Advice to avoid alcohol and tobacco use to:<br>a) pregnant teenage females	1	2	3	4	5
	b) pregnant unmarried women	1	2	3	4	5
	c) pregnant married women	1	2	3	4	5
7.	Advice on good nutrition, breast-feeding and delivery options:<br>a) teenage females	1	2	3	4	5
	b) unmarried women					
	c) married women					
**D2. PROVISION OF CARE DURING LABOUR**						
During the past month (30 days), how often did you provide the following care during labour?<br>Never or 0 days=1, Rarely or 1-2 days= 2, Sometimes or 3-9=3, Often 10-19 days= 4 and Very often >20 days= 5						
1)	Pelvic examination every 2 to 3hrs women	Never (1)	Rarely (2)	Sometimes (3)	Often (4)	Very often (5)
2)	Maternal heart rate and BP monitoring	1	2	3	4	5
3)	Fetal heart rate	1	2	3	4	5
4)	Fetal heart sounds	1	2	3	4	5
5)	Provide IV infusion	1	2	3	4	5
6)	Provide analgesics	1	2	3	4	5
**D3. PROVISION OF NEONATAL CARE**						
During the past month (MONTH/ 30 days), how often did you provide the following neonatal care?<br>Never or 0 days=1, Rarely or 1-2 days= 2, Sometimes or 3-9=3, Often 10-19 days= 4 and Very often >20 days= 5						
1)	Assessed the Apgar score components at 1 and 5 minutes(colour, heart rate, reflex responses, muscle tone, respiration) on a new-born	Never (0 days)	Rarely (1 or 2 days)	Sometimes (3 to 9 days)	Often (10 to 19 days)	Very often (20 or more days)
2)	Administered preventive agents (antimicrobial agents, Vitamin K) to a new-born	1	2	3	4	5
3)	Measured length, weight and head circumference of the new-born	1	2	3	4	5

**D4. KNOWLEDGE OF MATERNAL AND CHILD HEALTH SERVICES**				
The following statements are about maternal and child healthcare knowledge. Please tell me if you think the statement is true, false, or if you do not know.				
		True	False	I don't know
1)	In South Africa, married women are entitled to maternal and child care services more than others.	1	2	3
2)	Family planning service are a basic component of	1	2	3

	maternal and child health services.			
3)	In South Africa, all women have access to antenatal care services.	1	2	3
4)	Tetanus immunisation is not compulsory for a pregnant woman in South Africa.	1	2	3
5)	Advice on proper nutrition and breast-feeding is an essential part of educational counselling during pregnancy.	1	2	3
6)	Routine prenatal blood screening (CBC, HepB, HIV, syphilis) among pregnant women is mandatory.	1	2	3
7)	Routine prenatal physical examination (height, weight, BP, fetal heart sounds, size and position of the fetus) among pregnant women can be carried out only on the 3rd trimester.	1	2	3
8)	Iron supplements are necessary during pregnancy.	1	2	3
9)	Folate supplements should not be provided to a pregnant woman.	1	2	3
10)	It is okay for a pregnant woman to consume alcohol.	1	2	3
11)	Smoking is dangerous during pregnancy.	1	2	3
12)	It is important to detect HIV infection early in pregnancy.	1	2	3
13)	Early detection of non-communicable diseases (NCDs) is not necessary for a pregnant woman.	1	2	3
14)	Hypertension (eclampsia/pre-eclampsia) in pregnancy is not a threat to the fetus.	1	2	3
15)	Management of diabetes can resume only when the baby is born.	1	2	3
16)	PMTCT is only for the unborn baby.	1	2	3
17)	Child immunization should be carried out to babies born in hospital only.	1	2	3
18)	Healthcare workers are not obligated to monitor women for possible postpartum complications after the birth.	1	2	3

D6. SUBJECTIVE NORMS TOWARDS MATERNAL AND CHILD HEALTH SERVICES (these are norms among colleagues)

	**Please indicate how strongly you agree with the following statements:**	Strongly Disagree	Disagree	Neutral	Agree	Strongly Agree
1)	Most of your colleagues think that you should advise pregnant women to avoid alcohol and tobacco use	1	2	3	4	5
2)	Most of your colleagues think that you should provide good nutrition advice to a pregnant woman	1	2	3	4	5
3)	Most of your colleagues think that you should provide breast-feeding advice to pregnant women	1	2	3	4	5
4)	Most of your colleagues think that you should discuss delivery options with a	1	2	3	4	5

	pregnant teenage females					
5)	Most of your colleagues think that you should discuss delivery options with adult pregnant women	1	2	3	4	5
**D7. SELF-EFFICACY TO PROVIDE MATERNAL AND CHILD HEALTH SERVICES**						
**Please indicate how confident you are to provide the following services**		Not confident at all	Not confident	Unsure	Confident	Very confident
1)	How confident are you that you will be able to provide routine prenatal physical examination (height, weight, BP, fetal heart sounds, size and position of the fetus) to pregnant women?	1	2	3	4	5
2)	How confident are you that you will be able to provide routine prenatal blood screening (CBC, HepB, HIV, syphilis, diabetes) to pregnant women?	1	2	3	4	5
3)	How confident are you that you will be able to provide iron supplements to pregnant women?	1	2	3	4	5
4)	How confident are you that you will be able to provide Folate supplements to pregnant women?	1	2	3	4	5
5)	How confident are you that you will be able to provide Immunization for diphtheria, tetanus and pertussis to pregnant women?	1	2	3	4	5
6)	How confident are you that you will be able to advise pregnant women to avoid alcohol and tobacco use?	1	2	3	4	5
7)	How confident are you that you will be able to provide good nutrition advice to pregnant women?	1	2	3	4	5
8)	How confident are you that you will be able to provide breast-feeding advice to pregnant women?	1	2	3	4	5
9)	How confident are you that you will be able to discuss delivery options with a pregnant teenage female?	1	2	3	4	5

10)	How confident are you that you will be able to discuss delivery options with adult pregnant women?	1	2	3	4	5

D8. INTENTION TO PROVIDE MATERNAL AND CHILD HEALTH SERVICES

**Please indicate whether you intend to provide the following services**		Definitely not at all	Not confident	Unsure	Yes	Definitely yes
1)	The next time a pregnant woman needs routine prenatal physical examination (height, weight, BP, fetal heart sounds, size and position of the fetus), I intend to provide it.	1	2	3	4	5
2)	The next time a pregnant woman needs routine prenatal blood screening (CBC, HepB, HIV, syphilis, diabetes), I intend to provide it.	1	2	3	4	5
3)	The next time a pregnant woman needs iron supplements, I intend to provide it.	1	2	3	4	5
4)	The next time a pregnant woman needs Folate supplements, I intend to provide it	1	2	3	4	5
5)	The next time a pregnant woman needs Immunization for diphtheria, tetanus and pertussis, I intend to provide it.	1	2	3	4	5
6)	The next time a pregnant woman needs advice to avoid alcohol and tobacco use, I intend to provide it.	1	2	3	4	5
7)	The next time a pregnant woman needs good nutrition advice, I intend to provide it.	1	2	3	4	5
8)	The next time a pregnant woman needs breast-feeding advice, I intend to provide it.	1	2	3	4	5
9)	The next time a pregnant teenage female needs advice on delivery options, I intend to provide it.	1	2	3	4	5
10)	The next time a pregnant woman needs advice on delivery options, I intend to provide	1	2	3	4	5

THANK YOU FOR YOUR PARTICIPATION!
